# Female transactional sex workers’ experiences and health-seeking behaviour in low-middle income countries: a scoping review

**DOI:** 10.1186/s12889-024-20211-7

**Published:** 2024-10-09

**Authors:** Joseph Kwame Wulifan

**Affiliations:** Department of Geography, Faculty of Social Science and Arts, Simon Diedong Dombo, University of Business and Integrated Development Studies, Bamahu, Wa Ghana

**Keywords:** Commercial sex work, Prostitution, Transactional sex, Low-middle income countries

## Abstract

**Background:**

For a variety of reasons related to biology, behaviour, and environment, a subset of a population known as female sex workers (FSWs) or female transactional sex workers is at increased risk of health, depression, social stigma and access to timely and quality healthcare when needed. In low- and middle-income countries (LMICs), there was lack of understanding regarding the experiences and healthcare utilisation and behaviours, the health burden among them, their experiences, and how they access health care. Using Anderson’s behavioural model of health service utilisation as a framework, this review aimed to explore the experiences of, and healthcare seeking behaviours of female sex workers in low-and middle-income countries.

**Methods:**

Six relevant databases such as PubMed, Embase, Global Health, Scopus, Web of Science, and Google Scholar were searched for peer-reviewed research articles published between January 1990 and December 2023 that discussed female transactional sex work in low- and middle-income countries. Subject terms such as: low-and middle- incomes, sex workers (female and male), sexually transmitted infections (STIs) in the sex work industry, prostitution, commercial sex, and health-seeking behaviour were used for the databases search. Out of 6,135 articles that were retrieved for the study, 26 met the inclusion criteria. Of the total number of studies, four were reviews, eight were quantitative studies, six were qualitative studies, and two utilised mixed methods.

**Findings:**

Results from a thematic analysis of studies that combined quantitative and qualitative methods yielded six overarching themes.The study found that women engaged in sex work for different reasons – to fend for themselves (i.e., livelihood), self-employment and others do it for pleasure. However, force sex or unprotected which can lead to sexually transmitted infections, sexual abuse, job insecurity, were critical risks factors in engaging in sex work. These factors make them vulnerable to predators and health risks. It was found that sex workers were aware of the importance of seeking healthcare, and do make the efforts, however, crucial factors such as difficulty accessing healthcare and maltreatment by healthcare providers and social stigma disincentivises FSW health-seeking behaviours. Sexual workers reported discomfort disclosing their occupations because of the stigma and discrimination which further affects their regular health examinations and obtaining medically approved condoms from healthcare facilities.

**Conclusion:**

Complex challenges rooted in economic vulnerability, social marginalisation, and limited access to healthcare afflict female sex workers in LMICs. The maltreatment and stigma can potentially affect LMICs from achieving using health facility care, with potential implications on achieving the universal health coverage goals. Comprehensive, rights-based strategies that address structural injustices and empower these women to live healthier, more secure lives are necessary to address their special needs.

## Background

 Female transactional sexual behaviour is associated with public health problems worldwide and presents many conceptual challenges for researchers [1–3]. The term “female sex work” refers to a wide variety of situations and behaviours where sexual services are exchanged for monetary compensations or goods. This concept acknowledges the agency of women who work in the sex industry while also taking into account the intricate web of socioeconomic, legal, and cultural influences on their lives. Women predominantly perform transactional sex work, which involves exchanging sexual services for economic benefits, material products, or engaging in social outings in exchange for financial gain [4, 5]. Sex workers provide a range of sexual services, classified as either direct or indirect sex work [6, 7]. Direct sex work encompasses several activities, including indoor and outdoor prostitution, escort services, and the exchange of sex for a price. This typically involves genital contact and takes place in guesthouses, hotels, or brothels [6, 8, 9]. Direct sex labour refers to the act of offering sexual services, including sexual intercourse, oral sex, or other sexual acts, in return for monetary compensation or products. Contrarily, indirect sex work encompasses non-sexual activities connected to the sex business, like exotic dance, webcam performances, or escort services. While the focus may not be solely on sexual acts, it can also involve companionship [9, 10].People that are involved in commercial sex work are vulnerable to sexually transmitted infections (STIs) and often face violent acts from clients, pimps, police, and other individuals [1, 4, 9].

The health and well-being of female sex workers (FSWs) are greatly impacted by a number of issues and vulnerabilities that are specific to the profession in low- and middle-income countries (LMICs). Thus, FSW in low- and middle-income countries (LMICs) should not be overlooked as a minor aspect of the social and economic crisis. Governments are becoming more aware of the problems and dangers faced by sex workers and their health-seeking activities [4, 6–8]. Legal ambiguities, cultural stigmas, and socioeconomic volatility are common features of the sex industry landscape in low- and middle-income countries (LMICs), and they have a significant impact on the lives of female sex workers. Many FSWs turn to sex work as a way to make ends meet in the face of scarce economic possibilities, which are frequently caused by gender inequity, poverty, and a lack of education. These socioeconomic factors affect women’s behaviours related to obtaining health care as well as pushing them into prostitution. In LMICs, it was found that mental health illnesses are highly prevalent among FSWs. According to a systematic review, about 21.0% of FSWs suffer anxiety and 41.8% of them struggle with depression [11]. Numerous risk factors, including poverty, inadequate education, violence, substance abuse, and the stigma attached to sex work, are frequently connected to mental health conditions [11]. Beksinska and colleagues [12] noted that FSWs in LMICs engage in harmful alcohol use, with two-fifths reporting alcohol use and one-quarter daily use compared to 5.1% alcohol consumption among women in the general population [12].

In addition, the environment in which FSWs conduct their business often vary and with different experiences, thereof. According to a qualitative study conducted in Kampala, Uganda, FSWs meet clients online as well as in real-world settings like venues and outdoor areas. Due to the criminalisation of their profession, the women involved reported experiencing severe stigma, assault from customers and authorities, and difficulties getting access to healthcare. Those who used online venues faced extra dangers, including extortion and cybersecurity threats [13].

In urban versus rural settings, FSWs may have very different experiences. Though there is more competition and violence in urban areas; therefore, FSWs may have better access to clients and services [2, 11, 13]. On the other hand, the lack of clientele and restricted availability of quality healthcare services in rural areas may make FSWs more susceptible to health risks. Indeed, previous studies have demonstrated that FSWs do experience varying degrees of violence depending on their physical environment, including the presence of law enforcement agencies such as police and the general local community safety measures. Ironically, it was reported that increased Police Officers’ presence in some geographical locations resulted to more violence against FSWs, while less law enforcement agencies in other places, allowed FSWs customers or traffickers to use violence without consequences [2, 11, 13]. These security, socio-cultural characterisation of commercial or transactional sex work, have had dire health implications of female sex workers. Indeed, discrimination and stigma from communities and healthcare providers have disincentivised FSW from seeking healthcare.

However, the universal health coverage (UHC) principles are widely championed because a population’s healthcare-seeking behaviour (HSB) has a major impact on a country’s health status and, as a result, socioeconomic growth [14, 15]. ‘Health-seeking behaviour (HSB)’ involves individuals recognising symptoms, seeking help, and choosing healthcare providers or treatment methods. HSB refers to the behaviour of persons who either do nothing, delay taking action, or take action after understanding they are not in good health or have a specific health problem in order to discover the best option to restore their health [14, 16]. Notably, commercial sex workers in low- and middle-income countries, have a poor track record of seeking healthcare [4, 16].

It includes factors such as the type of provider, adherence to treatment, reasons for choosing a professional, and reasons for not seeking help. The environment’s characteristics significantly influence individuals’ decisions to seek healthcare, with specific characteristics determining their HSBs [15]. This decision-making process is influenced by personal beliefs, perceived illness severity, access to healthcare services, and socio-demographic variables like age, gender, and socio-economic status. Indeed, behaviours relative to healthcare seeking may be influenced by ‘self-determination’ and ‘self-care’ [15, 17, 18]. Female sex workers (FSWs) face numerous health risks, including higher rates of STIs, HIV, and reproductive health issues. Sex work often involve pressure, assault, and exploitation, making it difficult for them to access necessary care.

The literature has shown that many FSWs face significant barriers to accessing healthcare, preferring informal providers due to fear of stigma and discrimination. Studies show that up to 70% of FSWs avoid public health facilities due to discrimination, stigma and mistreatment concerns [19]. Indeed, in Kenya, 56% of FSWs accessed community health services, because they were more accessible and less stigmatizing than formal healthcare settings [19]. Consistent use of condoms by FSWs is linked to increased healthcare service seeking, with a meta-analysis showing a 65% higher likelihood of avoiding services due to fear of judgment or discrimination [12]. In Mumbai, it was found that 45% of FSWs have accessed healthcare services in the past year, mainly for reproductive health issues. However, many prefer private clinics due to fear of stigma [20]. Another 50% of FSWs in Nairobi, Kenya, sought healthcare for STIs in the past six months, but barriers like police harassment and stigma led to delays [11]. In Uganda, 40% of FSWs used mobile health services, but only 25% visited a healthcare facility for regular check-ups [11]. In South Africa, 70% of FSWs experienced violence, negatively impacting their health-seeking behaviour. Only 30% sought medical care after violent incidents, reflecting fear of further victimisation and stigma within healthcare settings [12]. To address intrinsic healthcare seeking behaviours issues, 60% of FSWs in São Paulo were given health education programs, which helped to increase their likelihood of seeking preventive healthcare services [19]. FSWs were also more likely to experience mental health problems like anxiety and depression, often due to stigma and violence. Barriers such as a lacklustre healthcare system and social perceptions about sex work, significantly impacted their health-seeking behaviours [17, 18, 20].

**Fig. 1:**
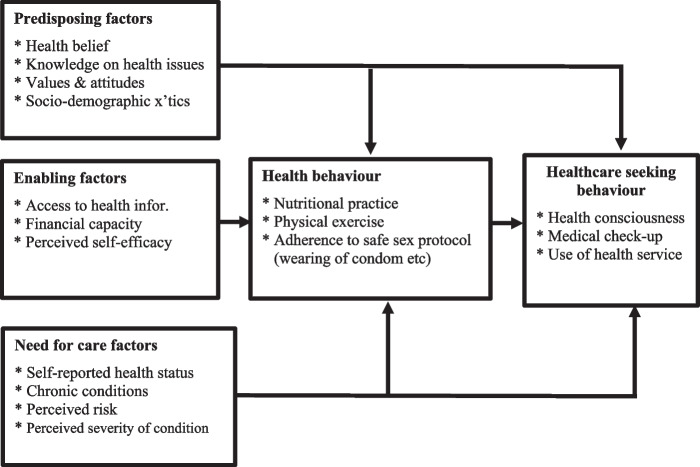
Health service utilization model. Source: Adapted from Saah; Andersen and Davidson [22–24]

This review draws on Anderson’s model of health-seeking behaviour Fig. 1, also known as Anderson’s Model of Health Services Utilisation [21], to explore female sex workers experiences and health-seeking behaviours in low-and middle-income countries. The model consists of three primary components: predisposing factors, enabling factors, and need factors [21]. The predisposing factors describe the tendency to use services. In the model, ‘predisposing’ factors, such as beliefs, social structure, and demographics, can influence a person’s propensity to seek health services. For female sex workers, these factors may include age, education, and ethnicity. Besides the ‘predisposing’ factors, Anderson’s model explains ‘enabling’ factors as the means available to use health services including personal and family resources (i.e., wealth status and social support) and community resources (i.e., residence and access to health resources). to include factors, such as financial resources, reliable transportation, and social support networks, facilitate or hinder health-seeking behaviour. Finally, ‘need’ factors, pertain to the female sex worker’s (FSWs) personal and contextual level’s evaluation of the severity of a sickness or health condition. ‘Need’ factors can be influenced by objective and subjective factors, include physical health issues like STIs, substance abuse, and mental health concerns. The severity of these health conditions, whether perceived or actual, influences their decision to seek medical attention. Overall, understanding and addressing these factors can help female sex workers better manage their health needs [21].

Thus, this study aim to explore their sex work experiences and health-seeking behaviours in low- and middle-income countries. This review provides a comprehensive understanding of female sex work in LMICs, addressing the unique challenges faced by sex workers. It highlights the complexities of healthcare utilization and the social, economic, and security threats to their livelihoods, thereby enabling the development of public health efforts and interventions [25, 26].

## Methods

### Design

To find relevant research, this review conducted a comprehensive mapping of studies that focused on commercial or transactional sex work and the health-seeking behaviours (HSBs) of female sex workers. The following steps proposed by Arksey and O’Malley’s [27], were followed. Arksey and O’Malley’s [27], framework prescribes five sequential stages in conducting scoping reviews; identifying the relevant studies, selecting the relevant studies, charting, collating, summarising, and reporting the results from the selected studies, as explained in the subsequent paragraphs. Additionally, certain components were incorporated from Pluye et al.‘s [28], framework for mixed method reviews [27–31], enhancing the overall analysis and reporting of the findings.

### Information sources

These searches were conducted using academic databases such as PubMed, Embase, Global Health, Scopus, Web of Science, and Google Scholar. In addition, the author conducted a thorough search for non-traditional sources of information, as well as examined the reference lists of all relevant studies that were found, in order to uncover any more research papers that align with our search criteria [27, 28]. The literature searches were conducted in November 2023 and updated in December 2023.

### Database search

A systematic search was conducted on published literature within these popular databases in the field health, psychology and social sciences. The following keywords and Medical Subject Headings (MeSH) terms were combined in the searches: “transactional sex work” OR “commercial sex work” “prostitute” OR “prostitution” OR “sex work” OR “sex worker” OR “female sex workers” OR “STIs” OR “health-seeking behavior” AND “low-middle income countries”.

### Eligibility (inclusion/exclusion criteria)

The study included studies published between 1990 and 2023. This was the period within which discussions on HIV/AIDS and STIs’ among female sex workers became prominent in LMICs, including Sub-Saharan Africa [32, 33]. It included studies targeting active reproductive ages (15–49) and excluded those primarily focusing on STIs rather than commercial sex or prostitution. The target population is often sexually active and at risk of STIs [32, 33]. Other article eligibility criteria include: must be quantitative, qualitative or mixed methods study. Article should be published in English Language. Article report findings of a study conducted in low-and middle-income country. Participants must in the study should be female sex workers, or health professionals who provide care to female sex workers. The article must be published in a peer-reviewed journal outlet. Non-peer reviewed journal articles, commentaries, editorials, letters written to editors or policy statements were excluded. For inclusion, studies must focus only on female sex workers and their potential linkage to the health services delivery system and health seeking behaviours.

### Charting, collating, summarising and reporting

**Fig. 2:**
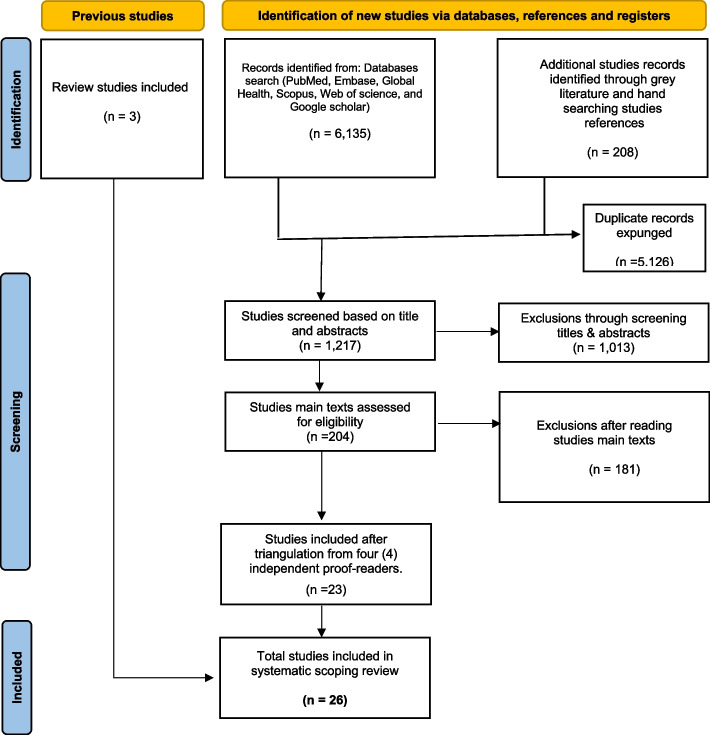
PRISMA flow diagram adopted for studies included in this systematic scoping review: Study identification, screening, exclusions and final inclusions after assessing studies full texts (Page et al., 2021)

Relevant components of the study aims, methods used and the findings were extracted and summarised. Out of 6,347 articles found using the database and manual search, 1,215 made it through the title and abstract screening process after duplicates were removed. After the screening, 202 studies were considered eligible for assessment, and 22 studies were ultimately selected after triangulation by three (3) separate reviewers. The final tally for the papers included in the analysis was 26 after screening and adding 3 prior review studies (Fig. 2).

The relevant information in the articles used in the study were independently extracted, examined, and synthesised relative to the experiences and healthcare-seeking behaviours of FSWs. Three colleagues with advanced expertise in review studies were engaged to assess and triangulate the findings obtained from the summaries of the findings, focusing on factors that resonate with the study’s objective. Both qualitative and quantitative data were obtained and presented using narrative synthesis to understand the factors [27, 28]. Manual extraction, coding, phrase/word search and reading through the summaries of the extracted data, generated recurring terms and codes. The iteration involved repeated reading of the data and codes. The codes showed coherent patterns of the experiences and healthcare utilisation of female sex workers. The patterns were reconciled and brought together to form themes. The themes were named incongruence with the Anderson’s model and the research objective. A synthesis and narrations of the findings were presented and supported with selected participant expressed views, for emphasis.

## Results

### Characteristic of included studies

The twenty-six articles included were published between 1990 and 2022 of which twelve articles were published in 2012, 2013 and 2015 [5, 9, 26, 34–43]. Articles included in this study covers many low-and middle-income countries. Specifically, four studies each were conducted in Ghana and Kenya, three from Nigeria, two each from Uganda, India and LMICs and the remaining one study each from China, Ethiopia, Nepal, Senegal, Gambia, Cuba, Malawi, Africa and South Africa (Fig, 3). Of these studies, nine were quantitative studies [8, 37–45], eight qualitative [4, 5, 9, 46–49], six mixed methods studies [9, 36, 44, 50–52] and three review studies [26, 35, 42] (Tables 1, 2 and 3).

**Fig. 3:**
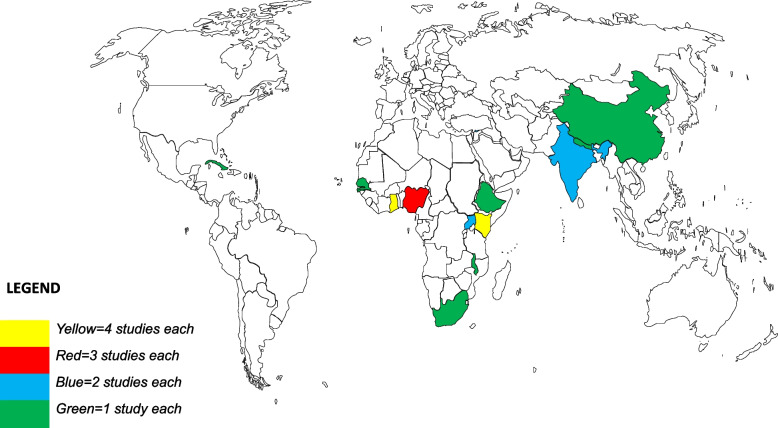
Geographic distribution of reviewed studies. This map provides an overview of the geographic distribution (shaded countries) of those studies in this review with a country specific focus. (Source: Author’s construct using the World map free template.net, 2022)

**Table 1:** Summary of mapped studies included in the systematic scoping review

**S/N**	**Author**	**Year**	**Country**	**Title**	**Objective**	**Publication type**	**Data collection**	**Analytical approach**	**Key findings**
**1**	**Baafour Opoku**	2015	Kumasi, Ghana	“It’s All About Making a Life”: Young Female Sex Workers Vulnerability to HIV and Prevention Needs in Kumasi, Ghana A Qualitative Study	1) Investigate knowledge about and perceptions of HIV in young FSW in the Kumasi Metropolitan area, 2) Explore their risk behaviours, and 3) Identify their most urgent prevention needs.	Qualitative study. Grey Literature (Report)	Female sex workers were recruited using a snowball sampling technique	Thematic analysis	Push factors included: familial poverty, leaving school, and inherited sex work. Pull factors included: friends, financial need, and lure of economic opportunity in Kumasi. Having no option emerged as a recurring theme.
**2**	**Baral S. et al**	2012	LMICs	Burden of HIV among female sex workers in low-income and middle- income countries: a systematic review and meta-analysis	Study aimed to assess the burden of HIV in female sex workers population compared with that of other women of reproductive age.	Systematic review	Database studies published between Jan 1, 2007, and June 25, 2011	Meta-analyses were done with the Mantel- Haenszel method with a random-effect model.	In 26 countries with medium and high background HIV prevalence, 30·7% (95% CI 30·2–31·3; 8627 of 28 075) of sex workers were HIV-positive and the odds ratio for infection was 11·6 (95% CI 9·1–14·8).
**3**	**Bekker et al**	2015	LMICs	Combination HIV prevention for female sex workers: what is the evidence?	To establish how a combining biomedical approaches with a prevention package, including behavioural and structural components as part of a community-driven approach, will help to reduce HIV infection in sex workers in different settings worldwide.	Quantitative study (Review)	HIV and sex workers Series infographic used. The literature review focused on HIV prevention programmes and interventions, and in particular those that focused on the female sex worker (FSW) population.	A model simulation based on the South African heterosexual epidemic.	This review gives evidence of an impressive array of already existing prevention modalities that can be combined and applied to reduce risk of HIV acquisition in FSW populations worldwide. Inadequate financing for FSW HIV prevention programming is a crucial reason why HIV prevention coverage remains so low.
4	**Boateng D. P**.	2016	Adum, Kumasi, Ghana	Health seeking behaviour among commercial sex workers in Adum in the Ashanti Region of Ghana.	The study sought to assess health-seeking behaviour among commercial sex workers in Adum in the Ashanti Region	Quantitative study. Grey Literature (MSc Thesis)	Structured questionnaires were administered to 140 female commercial sex workers in the Adum seaters.	Descriptive statistics and regression analysis	The study revealed that commercial sex business has clearly had a negative effect on the health of participants. 80.7% respondents indicated had Gonorrhea and 70.7% had Candidiasis.
**5**	**Choi S. Y. P. & Holroyd E.**	2007	Mainland China	The Influence of Power, Poverty and Agency in the Negotiation of Condom Use for Female Sex Workers in Mainland China	to assess barriers to consistent condom use in the context of transactional sex among female sex workers in mainland China.	Qualitative study	Data was collected by means of field observation of entertainment venues and in-depth interviews	Thematic analysis	Compared with other sex workers, street-walkers are less likely to use condoms with their clients, hold highly disadvantaged socioeconomic profiles and work in isolation
**6**	**Elmore-Meegan M. et al**	2004	Kenya	Sex Workers in Kenya, Numbers of Clients and Associated Risks: An Exploratory Survey	to find out where sex workers worked, the number of clients they had and the risks they were exposed to.	Qualitative study	In 2000 and 2001, in collaboration with sex workers who had formed a network of self-help groups, an exploratory survey was conducted among 475 sex workers in four rural towns and three Nairobi townships.	Thematic analysis	Most of the women (88%) worked from bars, hotels, bus stages and discos; 57% lived with a stable partner and almost 90% had dependent children. In the previous month, 17% had been assaulted and 35% raped by clients. Unwanted pregnancy was common; 86% had had at least one abortion.
**7**	**Gbagbo &Gbagbo**	2021	Ghana	Commercial sex work among university students: a case study of four public universities in Ghana	To Examined commercial sex work in selected public Universities in Ghana to inform policy and program decisions for safer sex at the universities in Ghana.	Exploratory-mixed- method design	Respondents were identified using purposive and snowballing techniques while semi-structured questionnaires and in-depth interviews were used for data collection between 2017 and 2019	Quantitative data were analyzed using Statistical Package for Social Sciences version 23 and qualitative data analyzed thematically.	Findings show that there is a proliferation of commercial sex work on university campuses in Ghana for financial, material, and emotional gains. Student sex workers have devised various strategies to combine academic work and sex work
**8**	**Gezie et al**	2015	North West Ethiopia	Time to unsafe sexual practice among cross border female sex workers in Metemma Yohannes, North West Ethiopia	To find out the time at which unsafe sexual practice starts and factors which determine the practice among female sex workers in North-west Ethiopia	Quantitative study	A total of 467 women who had been sex workers at least for three months prior to the resumption of the study were included. A structured and pre-tested questionnaire was used to collect data from July-August, 2010.	Descriptive statistics was used to explore the data, and the Extended Cox- Regression model was employed to identify the predictors of time-to- unsafe sexual practice.	The hazard of unsafe sexual practice increased by 3.0 % every month (p-value =0.040) due to problem- drinking. Those female sex workers with familiarized clients had a two-fold hazard of practicing unsafe sex compared to their counterparts (AHR = 1.94 95 % CI 1.49, 2.53).
**9**	**Ghimire et al**	2011	Nepal	Utilisation of sexual health services by female sex workers in Nepal	This study aimed to explore the factors associated with utilisation of sexual health services by FSWs in the Kathmandu Valley of Nepa	Mixed method	Mixed-method approach consisting of an interviewer administered questionnaire-based survey and in-depth interviews.	Multiple regression analysis (Quantitative data) Thematic Analysis (Qualitative data)	Survey completed with 425 FSWs, showed that 90% FSWs self-reported sickness, and (30.8%) reported symptoms of STIs. A quarter (25%) of those reporting STIs had never visited any health facilities especially for sexual health services preferring to use non-governmental clinics (72%), private clinics (50%), hospital (27%) and health centres (13%). Multiple regression analysis showed that separated, married and street- based FSWs were more likely to seek health services from the clinics or hospitals. In- depth interviews with 15 FSWs revealed that FSWs perceived that personal, structural and socio-cultural barriers, such as inappropriate clinic opening hours, discrimination, the judgmental attitude of the service providers, lack of confidentiality, fear of public exposure, and higher fees for the services as barriers to their access and utilisation of sexual health services.
**10**	**Gysels et al**	2002	Uganda	Women who sell sex in a Ugandan trading town: life histories, survival strategies and risk	This study investigated how women in a trading town on the trans-Africa highway in southwest Uganda become involved in commercial sex work, which factors contribute to their economic success or lack of success, and what effect life trajectories and economic success have on negotiating power and risk behaviour.	Qualitative study	Over the course of two years detailed life histories of 34 women were collected through recording open, in-depth interviews, the collection of sexual and income and expenditure diaries, visits to the women’s native villages, and participant observation.	Thematic analysis	Three groups of women were identified:(1) women who work in the back-street bars, have no capital of their own and are almost entirely dependent on selling sex for their livelihood; (2) waitresses in the bars along the main road who engage in a more institutionalized kind of commercial sex, often mediated by middlemen and (3) the more successful entrepreneurs who earn money from their own bars as well as from commercial sex. The three groups had different risk profiles.
**11**	**Ito et al**	2018	Senegal	The effect of sex work regulation on health and well-being of sex workers: Evidence from Senegal	Although FSW was legalized since 1969 to limit the spread of STIs, there is no evidence so far of its impact on FSWs' health and well- being. The paper aims to fill this gap by exploiting a unique data set of registered and unregistered Senegalese FSWs.	Quantitative	Data were collected from 320 registered and 310 unregistered FSWs over 21 years of age and living in Dakar suburbs in June and July 2015, which represents 15% of the estimated total FSWs in the region of Dakar (APAPS, 2011–2012). Registered FSWs were recruited by the midwife in charge of their medical follow-up.	Used propensity score match modeling.	Legalizing FSW and registering them has a positive effect on FSWs' health. However, we find that registration reduces FSWs' subjective well-being. This finding is explained by the fact that registered FSWs are found to engage in more sex acts, in riskier sex acts, have less social support from their peers, and are more likely to experience violence from clients and police officers.
**12**	**Izugbara S**	2012	Kenya	Client Retention and Health Among Sex Workers in Nairobi, Kenya	to interrogate motivations and strategies for recruiting and retaining regular male clients among female sex workers (FSWs).	Qualitative study	Data collection involved an assortment of qualitative techniques, namely ethnographic observation, guided dialogues, and in-depth individual interviews mainly with FSWs operating in bars and streets in Nairobi.	Thematic analysis	Client retention enabled sex workers to manage the risk of reduced marriage prospects, guaranteed them steady work, livelihoods, and incomes, and prevented their victimization and harassment. To retain clients, sex workers obliged them a great deal, pretended they had quit prostitution, and sometimes resorted to magical practices.
**13**	**Mahapatra et al**	2013	Karnataka, India	Factors Associated with Risky Sexual Practices among Female Sex Workers in Karnataka, India	The objectives of this study are to develop a summary measure of risky sexual practice and examine the factors associated with this among female sex workers (FSWs) in Karnataka, India.	Quantitative	FSWs were recruited using the two- stage probability sampling design.	Univariate, bivariate and multivariate analyses were performed.	About 51% of FSWs had engaged in risky sexual practice. The odds of engaging in risky sex were higher among FSWs who were older (35+ years) than younger (18–25 years), who were currently married than never married, who were in sex work for 10+ years than those who were in sex work for less than five years, and who had sex with 3+ clients/day than those who had sex with fewer clients.
**14**	**Matovu & Ssebadduka**	2012	Uganda	Sexual risk behaviours, condom use and sexually transmitted infection treatment-seeking behaviours among female sex workers and truck drivers in Uganda	An assessment of sexual risk behaviours, condom use and sexually transmitted infection (STI) treatment-seeking behaviours among truckers and female sex workers (FSWs) operating at 12 hotspots along two major transport corridors in Uganda.	Quantitative	Participants were identified through local contact persons: 261 truckers and 259 FSWs were interviewed.	Descriptive statistics was used to describe sexual risk behaviours, condom use practices and STI treatment-seeking behaviours among FSWs and truckers.	Ninety-four percent of FSWs and 87% of truckers reported condom use in the past month; however, only 21% of truckers and 45% of FSWs reported using condoms consistently during that time. More than half of truckers (n ¼ 261) and 77% of FSWs (n ¼ 259) reported that they suffered from STIs in the past year, and 93% of FSWs and 92.9% of truckers sought treatment for STIs in the past year
**15**	**Nkrumah W. K.**	2020	Cape Coast, Ghana	Nature and practice of commercial sex work in the Cape Coast metropolis of Ghana	The purpose of this study is to gain a deeper understanding of the issue of commercial sex work in the Cape Coast Metropolis.	Mixed Methods, Grey Literature (PhD thesis)	356 commercial sex workers and eight persons in key positions in key stakeholder institutions, all sampled from the Cape Coast Metropolis. Multi-stage sampling procedures were used for each category of participants:	Descriptive statistics & Thematic Analysis	Age and level of education had significant influence on the level of participation in commercial sex work. However, marital status and religion did not have any significant influence on the level of participation in commercial sex work.
**16**	**Obiageri O. A.**	2013	Nigeria	Causes and effects of commercial sex work among Akwa Ibom Girls: a study of youths in Uyo Local Government Area, Nigeria	The study seeks to find out the possible ways in which the problem of commercial sex work can be eradicated in the society.	Quantitative Grey Literature (Thesis)	Data was obtained through the primary and secondary sources. Instrument for data collection was questionnaire.	Descriptive statistics	Commercial sex workers are been categorized into various types, but the most commonness type of sex work is the child prostitution. There are various effects of commercial sex work in our society, ranging from HIV/AIDS, unwanted pregnancy, untimely death and STD
**17**	**Izugbara C. O**	2005	Nigeria	‘Ashawo suppose shine her eyes’: Female sex workers and sex work risks in Nigeria	Drawing upon qualitative research with 127 sex workers in Aba, Nigeria, the present paper explores sex work risks as they are constructed, expressed, and acted upon by sex workers themselves.	Qualitative Study	Qualitative research data was drawn with 127 sex workers in Aba, Nigeria.	Thematic Analysis	The sex workers were generally eloquent in expressing the idea that their work was particularly risky. They mentioned client violence and abuses, sexually transmitted infections, stigma and rejection, reduced prospects of marriage, police intimidation, and accelerated aging or physical degeneration as some of the risks in sex work.
**18**	**Pickering et al**	1993	The Gambia	Determinants of condom use in 24000 prostitutes/client contacts in the Gambia	To determine the factors that influence condom use among prostitutes and their clients in the Gambia	Quantitative	A cohort of 181 prostitutes were recruited, comprising all those working in any of the seven bars at any time during March 1989 to mat 1990.	Multiple logistic regression analysis	Data on the 24181 sexual contacts reported by the prostitutes indicated condom use varied according to type of partner (from 84% with clients to only 4% with regular partners). Condom use with clients varied according to location (from 91% in high-class bars to 59% in rural markets)
**19**	**Pope Cynthia**	2005	Havana, Cuba	The Political Economy of Desires: Geographies of Female Sex Work in Havana, Cuba	It examines the physical, social, and moral spaces in which sex work takes place and teases out some of the more salient power relations involved in creating and maintaining these spaces.	Qualitative study	The fieldwork took place in Havana in the summer of 1998, one year in country from 1999-2000, and continuing research in the summers of 2002 and 2003. Of the 225 total interviews, 38 (37 women and one man) were used for this article	Thematic Analysis	The study highlights race and class issues that many people think have been eradicated by Revolutionary ideology; and it shows how women’s bodies, and not just sex workers’ bodies, have been commodified for personal, and even national, economic gain.
**20**	**Savva Helen**	2013	South Africa	Factors Associated with the Utilization of Health Services by Female Sex Workers in South Africa	The overall objective of this study was to examine factors that influence FSWs’ utilization of and satisfaction with health services in four settings in South Africa for the period April to August 2010.	Mixed Methods, Grey Literature (MPH thesis)	This is a secondary analysis of data from a cross-sectional study on sex work in South Africa. For this study, the population was limited to self-identified FSWs who were of any nationality and older than 18 years.	Used Bivariate and multivariate analysis for Quantitative data while thematic analysis was used for qualitative data	More than half of the participants (n=1,909) had used health services in the month preceding the study with over half of those opting for public health facilities. Independent variables that influenced health services utilization included town of residence, provincial birthplace, having adult dependents, and average number of clients in the past week (aOR 2.39, 95% CI 1.37-4.15).
**21**	**Scheibe et al**	2012	Africa	HIV prevention among female sex workers in Africa	To discuss progress, opportunities and barriers to building UNAIDS’ three pillars for effective HIV prevention measures for sex workers in Africa, as outlined in this guidance note (UNAIDS 2012).	Review	Following UNAIDS’ three pillar approach to HIV prevention and sex work	presentation of an overview of current opportunities, barriers and suggestions to improve HIV prevention policy and programming for sex work in Africa.	Universal access to a comprehensive package of HIV services is the first pillar. Reproductive health commodities; voluntary and anonymous HIV counselling and testing; treatment of sexually transmitted infections, HIV and opportunistic infections; harm reduction for substance use and psychosocial support services make up the recommended package of services.
**22**	**Scorgie et al**	2013	Kenya, Zimbabwe, Uganda & South Africa	‘We are despised in the hospitals’: sex workers' experiences of accessing health care in four African countries	To explore a detailed understanding of barriers to accessing care that would optimize design of improved services for Sex workers.	Review	In this study, trained sex workers conducted 55 in-depth interviews and 12 focus group discussions with 106 female, 26 male and 4 transgender sex workers across 6 urban sites in Kenya, Zimbabwe, Uganda and South Africa.	Thematic Analysis following an interpretive framework.	Participants cited numerous unmet health needs, including diagnosis and treatment for sexually transmitted infections and insufficient access to condoms and lubricant. Denial of treatment for injuries following physical assault or rape and general hostility from public-sector providers was common. Resources permitting, many sex workers attended private services, citing higher quality and respect for dignity and confidentiality.
**23**	**Sekoni et al**	2013	Nigeria	Sexually Transmitted Infections: Prevalence, Knowledge and Treatment Practices among Female Sex Workers in a Cosmopolitan City in Nigeria	This study sought to assess the prevalence, knowledge and treatment practices of sexually transmitted infections among brothel based female sex workers.	Quantitative -Descriptive cross sectional	Three hundred and twenty three consenting female sex workers were surveyed using pre tested, interviewer administered questionnaires.	Descriptive analysis	More than half of the respondents (54.2%) had poor knowledge of symptoms of sexually transmitted infections. Only 13.9% were aware that sexually transmitted infections could be asymptomatic. The self-reported prevalence of symptomatic sexually transmitted infections was 36.5%. About half of those with sexually transmitted infections sought treatment in a hospital or health centre while 32.5% from a patent medicine vendors.
**24**	**Sutherland et al**	2011	Kenya	Contraceptive needs of female sex workers in Kenya – A cross-sectional study	The objective of this study of FSWs in Kenya is to document patterns of contraceptive use and unmet need for contraception.	Mixed method	This research surveys a large sample of female sex workers (N ¼ 597) and also uses qualitative data from focus group discussions.	Descriptive analysis (Quantitative data) and Thematic analysis (Qualitative data)	The study found a great reliance on male condoms, coupled with inconsistent use at last sex, which resulted in a higher potential for unmet need for contraception than the elevated levels of modern contraceptives might suggest. Dual method use was also frequently encountered in this population and the benefits of this practice were clearly outlined by focus group participants.
**25**	Vijayakumar et al	2019	India	Sex Work, Marginalization, and Activism in India	Commentary on sex work in India	Qualitative (Commentary)	The commentary sought to explore and expand Benoit et al.’s (2018) target article which aims to take a global approach to sex work, ultimately arguing that sex work constitutes a form of exploited labor in “neoliberal capitalist societies.”	Thematic	Sex workers’ lives are layered, and they wear multiple identities—often they are migrants; sometimes they are slum dwellers; they work as construction workers, vendors, and factory workers; and they are parents and partners.
**26**	**Walden et al**	1999	Malawi	Measuring the impact of a behaviour change intervention for commercial sex workers and their potential clients in Malawi	An evaluation for impact f a peer-education HIV/AIDS prevention programme for bar-based sex workers and their potential clients (long-distance truck drivers) in Malawi	Mixed methods	Structured questionnaires and focus group discussions.	Descriptive analysis (Quantitative data) and Thematic analysis (Qualitative data)	The results showed that in the active districts, the presence of sex worker peer educators led to a increase in condom use with paying partners (90.3 compared to 66.7 and 76.3% in the two other groups— non-active and average) and increased condom distribution.

**Table 2:** Summary of Factors

**Predictors of commercial sex trade in LMICs**												
**S/N**	**Author**	**Factors that precipitate commercial sex trade**										
		**Socioeconomic profile of sex worker**	**Entry into sex work**	**Major barriers to condom use**	**Exposure to physical & sexual voilence**	**Determinants of commercial sex woeker's service**	**Mode of operations (modus operandi)**	**Carrer Dev't**	**Organization of sex work**	**Sustainability**	**Exiting factors**	**Challengenges/Consequences**
1	Baafuor Opoku (2015) [53]	✓	✓		✓			✓			✓	✓
2	Baral et al (2012) [35]							✓				✓
3	Bekker et al (2015) [37]		✓	✓	✓			✓	✓	✓	✓	
4	Boateng D. P. (2016) [8]	✓										
5	Choi & Holroyd (2007) [46]	✓	✓	✓	✓			✓			✓	✓
6	Elmore-Meegan M. et al 2004) [47]	✓	✓	✓	✓			✓			✓	
7	Gbagbo & Gbagbo (2021) [9]	✓	✓	✓	✓	✓	✓	✓	✓	✓	✓	✓
8	Gezie et al (2015) [38]	✓	✓	✓	✓	✓						✓
9	Ghimire et al (2011) [50]	✓	✓	✓			✓	✓	✓	✓	✓	✓
10	Gysels et al (2002) [54]	✓	✓	✓	✓	✓	✓	✓	✓		✓	✓
11	Ito et al (2018) [44]	✓	✓	✓	✓	✓	✓	✓	✓		✓	✓
12	Izugbara (2012) [34]		✓	✓	✓	✓	✓	✓	✓	✓	✓	✓
13	Mahapatra (2013) [39]	✓	✓	✓	✓	✓	✓	✓	✓	✓	✓	✓
14	Matovu & Sebadduka (2012) [40]	✓	✓	✓	✓	✓	✓	✓	✓	✓	✓	✓
15	Nkrumah W. K. (2020) [10]	✓	✓	✓	✓	✓	✓	✓	✓	✓	✓	✓
16	Obiageri O. A (2013) [55]	✓	✓	✓	✓	✓	✓	✓	✓	✓	✓	✓
17	Izugbara C. O. (2005) [48]	✓	✓	✓	✓	✓	✓	✓	✓	✓	✓	✓
18	Pickering et al (1993) [45]	✓	✓	✓	✓	✓	✓	✓		✓		✓
19	Pope Cynthia (2005) [4]	✓	✓	✓	✓	✓	✓	✓	✓		✓	✓
20	Savva Helen (2013) [36]	✓	✓	✓	✓	✓	✓	✓	✓		✓	✓
21	Scheibe et al (2012) [42]	✓			✓		✓			✓		✓
22	Scorgie et al (2013) [26]	✓	✓	✓	✓	✓	✓	✓	✓	✓	✓	✓
23	Sekoni et al (2013) [43]	✓	✓	✓	✓	✓	✓	✓	✓	✓	✓	✓
24	Sutherland et al (2011) [51]	✓	✓	✓	✓	✓	✓	✓	✓	✓	✓	✓
25	Vijayakumar et al (2019) [49]	✓							✓			✓
26	Walden et al (1999) [52]	✓							✓			✓

**Table 3:** Summary of Factors Affecting Health Seeking Behaviour (HSB) of prostitutes

		**Predisposing factors**						**Enabling factors**					**Need factors**		** Health seeking behaviour**		
		**Socio-demographic**															
		**Age**	**Gender**	**Marital Status**	**Occupation**	**Religion**	**Cultural**	**Household wealth**	**Educational**	**High fee/Insurance**	**Access to facility**	**Woman’s Autonomy**	**Illness stutus**	**Illness sevierity**	**Type/facilty**	**Check-up**	**Service use**
1	Baral et al (2012) [35]	✓	✓		✓				✓				✓	✓			
2	Boateng D. P. (2016) [8]	✓	✓	✓	✓	✓		✓	✓		✓		✓	✓	✓	✓	✓
3	Baafuor Opou (2015)	✓	✓	✓	✓	✓	✓	✓			✓		✓			✓	✓
4	Bekker et al (2015) [37]	✓	✓	✓	✓	✓	✓	✓			✓		✓			✓	✓
5	Choi & Holroyd (2007) [46]	✓	✓	✓	✓	✓	✓	✓	✓			✓					✓
6	Elmore-Meegan M. et al (2004) [47]		✓	✓	✓	✓	✓		✓			✓		✓		✓	✓
7	Gbagbo & Gbagbo (2021) [9]	✓	✓	✓	✓	✓	✓	✓	✓					✓		✓	✓
8	Gezie et al (2015) [38]	✓	✓	✓	✓	✓		✓						✓		✓	✓
9	Ghimire et al (2011) [50]	✓	✓	✓	✓	✓	✓	✓		✓	✓		✓		✓		✓
10	Gysels etal (2002) [54]	✓	✓	✓	✓	✓	✓	✓	✓	✓	✓	✓	✓	✓	✓	✓	✓
11	Ito et al (2018) [44]	✓	✓	✓	✓	✓	✓	✓	✓		✓	✓	✓	✓	✓	✓	✓
12	Izugbara (2012) [34]	✓	✓	✓	✓	✓	✓	✓	✓		✓	✓	✓	✓	✓	✓	✓
13	Mahapatra (2013) [39]	✓	✓	✓	✓	✓	✓	✓	✓				✓	✓	✓	✓	✓
14	Matovu & Ssebadduka (2012) [40]	✓	✓	✓	✓	✓	✓	✓	✓	✓	✓	✓	✓	✓	✓	✓	✓
15	Nkrumah W. K. (2020) [10]	✓	✓	✓	✓	✓	✓	✓	✓	✓	✓	✓	✓	✓	✓	✓	✓
16	Obiageri O. A (2013) [55]	✓	✓	✓	✓	✓	✓	✓	✓	✓	✓	✓	✓	✓	✓	✓	✓
17	Izugbara C. O. (2005) [48]	✓	✓	✓	✓	✓	✓	✓	✓	✓	✓	✓	✓	✓	✓	✓	✓
18	Pickering et al (1993) [45]	✓	✓	✓		✓		✓	✓	✓	✓		✓				✓
19	Pope Cynthia (2005) [4]	✓	✓	✓	✓	✓	✓	✓	✓	✓			✓		✓	✓	✓
20	Savva Helen (2013) [36]	✓	✓	✓	✓	✓	✓	✓	✓	✓	✓	✓	✓	✓	✓	✓	✓
21	Scheibe et al (2012) [42]	✓			✓			✓					✓		✓	✓	✓
22	Scorgie et al (2013) [26]	✓	✓		✓	✓	✓				✓	✓	✓	✓	✓	✓	✓
23	Sekoni et al (2013) [43]	✓	✓	✓	✓	✓	✓	✓	✓	✓	✓	✓	✓	✓	✓	✓	✓
24	Sutherland et al (2011) [51]	✓	✓	✓	✓	✓	✓	✓	✓	✓	✓	✓	✓	✓	✓	✓	✓
25	Vijayakumar et al (2019) [49]																
26	Walden et al (1999) [52]	✓								✓	✓	✓	✓	✓	✓	✓	✓

### Demographic and socio-economic profile of sex workers

Demographics and socioeconomic variables commonly identified and discussed in these studies included age, marital status, level of education, family background, ethnicity, religious backgrounds and poverty. In 80.8% (*n* = 21) of the studies reviewed, the women entered sex work after turning 15 to18 years. In 11% of the studies, commercial or transactional sex was started by ages 12 and 14 with the majority of women in the included studies, engaging in FSW within the ages of 18–35 years [5, 8, 9, 38, 39, 44, 46–48, 54]. Most (92%, *n* = 24) of the studies show that FSWs have had at least some form of formal education, had attained post basic level education or had completed high school [4, 5, 8, 9, 26, 42, 44, 46, 47, 51, 52].

### Factors that influence entry into sex work

In 19 (73.1%) studies participants identified factors that led them to female transactional sex work. FSWs indicated that the primary factor influencing their decisions to work in the area of commercial sex was that, they were from poor financial background, had no job, did not have any identifiable sources of income and found it difficult to fend for themselves [5, 8–10, 26, 34, 37–41, 43–48, 51].

Respondents in other studies involving FSWs in Nigeria and others in public Universities in Ghana, provided a list of reasons for entering into the sex work industry that extended beyond money or material benefits. A subset of participants in the study provided the following explanations: *“I’m from a very rich home and my friends are surprised to know that I’m in this commercial sex business with them. I don’t need money from the men I sleep with*,* but good sex*,* which makes me relax*,* emotionally stable and puts me in a good mood to study for my exams. I am not usually satisfied by one man but multiple sexual partners (Female*,* undergraduate)”* [9]. Another respondent indicated that “*I sometimes beg and pay guys to have sex with me in my hostel. In situations when this becomes a challenge*,* I step onto the street with the intention not to make money but to satisfy myself by having sex with multiple men in brothels. I’m gainfully employed on study leave but not interested in any man for a long/ marital relationship (Female*,* postgraduate student)”* [52, 56].

### Health risks and vulnerabilities in female sex work

From the analysis, female sex workers (FSWs) revealed that the majority of male clients exhibit resistance in regards to condom usage prior to engaging in sexual intercourse. The vulnerability of younger sex workers was heightened by their inexperience and lack of proficiency in bargaining, leading to difficulties in advocating for condom use during sexual encounters. FSWs also mentioned issues of vulnerability to extortion by state security personnel such as Police Officers, who sometimes would abuse sex workers physically, and demand unprotected sex. Most cultures in low-and middle-income countries (LMICs) frown upon transactional sex work. Thus, sex workers commonly reported discomfort discussing their profession, accessing sexually transmitted infections (STIs) testing, and when they need approved and affordable condoms from the local health clinics [4, 5, 9, 10, 36–40, 44, 46–52, 54, 57, 58]. Independent research findings indicated that the risks of engaging in transactional sex work involved not only violence or embarrassment, but also the high risks of contracting sicknesses. For instance, FSW in Havana in Cuba, recounted one of the most distressing encounter when a non-native individual adamantly refused to use sexual protection, although she objected to it [4]. Following her compliance with the requests and spending the entire night with him, he reneged on his obligation to compensate her [4]. As the financial contribution from a casual partners increased, it became increasingly challenging for women to assert their request for condom usage. Citing a specific occurrence in Uganda, a woman stated [54]: *‘I didn’t ask him to use condoms. I felt like I couldn’t*,* he had given me good money.’*’ Others do not even try to convince their casual partners because they desperately need the money. *“Some men refuse to use a condom and I accept because I don’t have anything to eat.’’* [54]. A respondent in a study in Kintampo municipality in Ghana reveals, “*there are situations when we become pregnant or get some sexually transmitted diseases. This is why we charge higher if a customer wants raw sex or in the event that a condom gets bursts in action so that we can quickly buy medications to prevent pregnancy and any sexually transmitted diseases (Female*,* postgraduate student)*. A respondent in a similar study noted that *“it’s sometimes difficult to avoid unprotected sex in this business no matter how hard we try”. She went further to add that*,* “there are situations where some customers will start fucking you with a condom on mutual agreement but will later remove the condom and forcefully fuck you especially when the man is on top of you”.* A respondent in a study in Cape Coast, Ghana, stated that, “*other customers (men) are able to negotiate and give additional money or even double the price to enable us accept unprotected sex………*,* others also become so aggressive if we refuse them unprotected sex*,* hence they physically and sexually abuse us” (Female undergraduate student)* [52].

### Exposure to physical and sexual violence

Several studies have revealed dangers associated with violence or sexual assault perpetrated by clients. The circumstances that are thought to increase the vulnerability of female sex workers (FSWs) to violence include visiting a client’s residence, engaging in nocturnal sessions, and meeting unfamiliar individuals at unknown locations [5, 36–49, 51, 54]. Besides these, it was reported that condom bursts during sexual intercourse was also a matter of grave concern. The studies have documented that this occurrence is attributed to either unintended consequences of vigorous sexual activity or deliberate harm caused by male customers [9, 10]. FSW were more worried about their health because some of issues they face involved high-profile persons and personnel of security agencies, which sometimes exposed them physical abuse, coercion for unprotective sex. Some FSWs were refused payment of agreed fees for the services provided. This was reported in many countries including Mainland China, Ghana, Nigeria, India, The Gambia, Kenya, Senegal [4, 5, 9, 10, 36–49, 51, 54].

### Mode of operation (modus operandi)

The iteration of the results show that FSWs visit drinking bars and night clubs in and around urban areas at night in search of customers, whilst others operate in rented rooms (i.e. hotels, guest houses, and brothels). There were other categories such as those who provide escort services to clients. These categories are highly paid female sex work and involve sexual encounters by appointment with an exclusive client. Such FSWs leave their mobile phone contacts and pictures in hotels and guest houses in, and around their respective areas of operations, to be contacted by prospective customers in Mainland China, The Gambia, Senegal, Nigeria and similar LMICs [4, 5, 9, 10, 36–46, 48, 49, 51, 54]. Indeed, sex workers reportedly collaborate with taxi drivers, who connect them to customers and arrange pick-ups and drop-offs at specific locations and times in exchange for fees. Movie halls in and around urban areas were also emerging as brothels. More recently, it was found that some students who engage FSW could provide services for clients using their rooms in the student hostel facility. Thus, such students convert their hostel rooms into brothels. Several reliable sources indicated that “*there are various joints*,* drinking bars and movie houses around this university that you can find student sex workers*,* particularly at night. I can take you there if you want to see for yourself”* (Taxi drivers) [9].

### Challenges and consequences of fsws

Studies maintain that once they are selling sex, they are also trying to avoid abuse from clients and law enforcement agencies, such Police Officers. It was found that FSWs sought love and to build a future. Therefore, FSW indulge in romantic relationships with young men without prior medical screening for health status including sexually transmitted infections [5, 8–10, 30, 34]. Indeed, the results show that FSWs often do risks into engaging in unprotected sex and sometimes provide free sex services with the hope of building intimacy with such preferred male clients. FSWs reportedly had babies with male clients, who often do not accept responsibility of the pregnancy and childcare. FSWs in such situations have had to send their children (from such unfortunate situations) to their home villages for family to support with childcare or resort to single parenting [5, 8–10, 30, 34]. Other consequences were psychosocial, customers “pushing the boundaries” and future aspirations. Outside of this sphere of human relationships, were the cultural, economic, and policy environments that can both facilitate, thwart and restrict efforts to help these women stay safe within the profession or exit the sex work [5, 8–10, 30, 34, 46, 48, 50, 52].

### Factors influencing health-seeking behaviour of fsws

Analysis of factors influencing health-seeking behaviours (HSBs) was based on Andersen’s behavioural model of healthcare utilisation [21, 57]. Despite some criticisms of Anderson’s model for neglecting sociocultural dimensions and interactions, as well as failing to consider the social construction of need and the importance of enabling resources in preventing service use as predisposing factors, it was deemed relevant to this study on commercial sex work among female sex workers [58, 59]. The reason for this is that its principles align with this research and has the structure to guide in data extraction and presentation of findings relative to healthcare-seeking behaviour at both the FSW and community levels. Andersen’s model suggests that health-seeking behaviour of individuals is a function of three groups of factors; predisposing, enabling and need [21, 60–63].

#### Predisposing factors

Andersen indicates that although predisposing and enabling factors are necessary for health service utilisation, they are not sufficient explanations for “actual use” of healthcare. “Actual use” is initiated by need, which might arise as a result of level of the health condition [21, 57, 60, 64]. Socio-demographic variables of FSWs that motivated health-seeking behaviour were age, marital status, education level, occupation and religion [5, 8–10, 30, 34, 46, 50, 52]. In terms of education, low educational attainment among FSWs in countries like Ghana, Nigeria, Kenya, India and other LMICs limits their understanding of health issues and available services. Specifically, FSWs who had attained higher education levels were more likely to utilize healthcare services effectively, as they understand STIs preventive measures and pre-exposure prophylaxis (PrEP) uptake and have better access to information [5, 8–10, 30, 34, 46, 50, 52].

#### Enabling factors

In this study, enabling factors at the female sex worker (FSW) level included wealth and income at the her disposal to cover the cost of assuming positive health-related lifestyles such as good nutrition, physical exercising, wearing of condom, engaging in other safe sex practices and good personal hygiene [64–66]. Other broad range of factors such as travel time to the health facility, the means of transportation, and waiting time for healthcare, were found to influence HSBs. In addition, health education and promotion as well as outreach programmes and health policies relevant factors that influenced FSWs healthy behaviours and subsequent health service utilisation [4, 8–10, 36, 39, 41, 45–48, 51].

#### Need factors

At the individual FSWs level, the model differentiates between perceived need for health services, which refers to how individuals perceive and experience their own health status (such as self-rated health, functional state, and illness symptoms), evaluated need, which involves objective measurements of FSWs’ health status, health screening, and need for health care [39, 43, 57, 58, 60, 65, 66]. FSWs often distinguish between several population health indicators, such as the rate of sexually transmitted infections (STIs) among commercial sex workers, death rates, and overall incidence and prevalence of sickness and deaths associated with sex work at both national and local levels. Younger FSWs in Ghana, Kenya, Nepal, India, South Africa, Uganda and Nigeria prioritised financial needs over health, leading to lower healthcare utilisation [10, 26, 36, 48, 49, 67]. Older workers, with more experience, were more proactive in seeking healthcare, including STI screenings and HIV testing. Furthermore, comprehensive assessments of community well-being, which encompass epidemiological measures of sexually transmitted infections (STIs), HIV/AIDS illness rates, and death rates, had an impact on FSWs’ tendencies to seek healthcare as reported in Argentina, albeit the finding is consistent with general healthcare utilisation in many LMICs [22, 58, 66].

The enhanced healthcare seeking behaviour among FSWs can be attributed to their apprehension of developing the intricate type of sexually transmitted infections (STIs) and their increased awareness about health, as shown in studies in Kenya, Ghana, Burkina Faso, and other LMICs [8, 9, 35, 51]. Two factors contributing to the improvement of care seeking behaviour were identified: increased health consciousness and regular self-reported medical check-ups among FSW in Ghana and Mainland China [5, 8, 46]. Some female sex workers (FSWs) promptly sought treatment upon experiencing symptoms, which indicates commendable behaviours exhibited by the sex workers in brothels in Ghana [8]. Streetwalkers, in comparison to other sex workers, exhibit lower condom usage with customers, possess significantly disadvantaged socioeconomic backgrounds, and operate in solitude. The primary obstacles to condoms’ usage or enforcing sexual protection norms, were associated with financial disadvantages and the risk of harm posed by customers. Female sex workers who operated within entertainment establishments were constrained in using condoms because customers were mostly drunk at the time of providing the services, existing unfavourable pricing regimes, and the level of acquaintance and intrinsic intents towards male clients, Nigeria, China, Ghana, Kenya and India [8–10, 34, 35, 46, 48, 51, 68]. Indeed, findings from Nigeria and Ghana show have shown that alcohol consumption decreases condom negotiation capabilities among sex workers, with 73% reporting client refusal to use condoms, often exacerbated by intoxication [8, 9, 48].

### Barriers to health-seeking behaviour

Two articles reported that women engaged in street-based sex work, whether separated or married, were more likely to seek health services from clinics or hospitals, when compared to those who were not or never married [9, 50]. Three studies conducted with female sex workers (FSWs) in Ghana and Nepal revealed that FSWs identified various barriers to accessing and utilising sexual health services [8, 10] 51]. These barriers included; inconvenient clinic hours, discrimination, judgmental attitudes from service providers, a lack of confidentiality, a fear of public exposure, and higher fees for services in Nepal and Ghana [8, 10, 50]. The prevalent infections among female sex workers (FSWs) included syphilis, gonorrhoea, candidiasis, and malaria [5, 8, 12, 26, 36, 43]. In three different studies in South Africa and Ghana, the primary motivation for seeking healthcare was the need for treatment for HIV, sexually transmitted illnesses, and tuberculosis. Besides these, adherence to antiretroviral therapy (ART) was low due to depression and stigma [19]. Counselling and assistance were the least frequently mentioned factors. Female sex workers who utilised health facility reported receiving satisfactory care among sex workers in Ghana and South Africa [5, 8, 36]. Alcohol intake among FSW was a crucial barrier to healthcare utilisation in LMICs, with attendant negative implications on their health outcomes [12].

## Discussion

The study aimed to synthesize existing findings on female sex workers experiences, survival strategies, and health-seeking behaviours in low- and middle-income countries. The findings revealed that female sex workers had a high level of formal education, were primarily between the ages of 12 and 35, were either gainfully employed in other sectors or unemployed, and had financial needs. Female sex workers (FSWs) who were married or separated showed an increased likelihood of seeking healthcare. However, FSWs faced challenges in seeking healthcare due to derogatory and discriminatory behaviours towards them, high healthcare fees, and discrimination and maltreatment from providers. Generally, the majority of women involved in transactional sex work experience various degrees of vulnerability. Thus, FSWs’ increased awareness of health concerns and their desire to protect themselves from HIV/AIDS and other sexually transmitted infections (STIs) motivated them to seek healthcare. Some FSWs engaged in the act to earn a living, while others primarily engaged in sex work for the fun of it.

The majority of the studies examined indicate that most women engaged in sex work at the age of 12 or older had received some level of formal education. These women faced a lack of opportunities to earn income, and many considered dropping out of school as a pivotal moment in their lives, which aligns with existing literature [54, 67, 69, 70]. Contrary to the notion that financial incentives alone drive women to engage in sex work, other research conducted in Canada and the UK challenges this perspective. Participants in these studies engaged in the sex trade due to financial constraints, but only as a final recourse after exhausting all other employment opportunities [67, 70]. This study revealed that some FSWs participated in the sex work for their own enjoyment, even among postgraduate students who had no intention of entering into marriage [48, 54]. The findings suggest that sex work was not wholly a job or trade, since not all aimed to benefit financially. While a mix of reasons exist for engaging in transactional sex work, the findings present substantial challenges.

First, male clients’ non-adherence and prioritisation of heath protection. Crucial concerns of their health was high, due to clients’ refusal, and deliberate uncooperative attitude towards condom usage during sexual service provision, and incidents of violence. It is worth noting that clients’ refusal to cherish and use sexual protection was closely linked to ‘power and control imbalances’ in the transactional sex work business, as well as the ‘terms’ of ‘contract’ between commercial sex workers and their male clients. From the results, the male clientele tends to exert more dominance in the entire negotiation process. In this context, the use of condoms during transactional sexual interactions is an essential aspect. A quick scan of the literature demonstrates that unprotected sex exposes the lives of FSWs and their clients, to STIs including HIV. Indeed, there was high HIV prevalence in the sex work industry. For example, in 2012, the World Bank report on the health of sex workers showed that across all regions, HIV prevalence was 11.8%, and 36.9% in sub-Saharan Africa, with Malawi (70.7%), South Africa (59.6%), Kenya (45.1%), Rwanda (24.0%) [71], . A 2020 survey in Iran revealed a low HIV prevalence of 1.6% [72]. More recently, a relatively lower rates of HIV prevalence was found in other LMICs in Eastern Europe, where a rate of 7.5% was recorded, Guyana (6.5%), Brazil (5.3%), India (2.2%) and 7.5% in Ukraine [73]. However, this shows that stringent policy, health education, and legislations were implemented and enforced to ensure the low risks of HIV transmissions. It was also noted that results of increased healthcare utilisation in those countries [73]. The majority of female sex workers lacked the ability to negotiate safe and respectful heterosexual relationships, including vaginal and anal intercourse, and often had limited control over their own bodies, as supported by previous research [10, 17, 20, 74]. The literature has generally firmly established the connection between intimate FSWs-client violence and adherence to condom usage. These concerns suggest that sex workers may prioritize the immediate danger or fear of violence over efforts to enforce condom use with clients, particularly in situations where violence against them was widespread and inadequately addressed [40, 42, 43].

Second, health and bodily security were crucial findings from literature. The findings have shown that FSWs were susceptible to multiple problems of health risks, violence and physical harm. Indeed, concerns relative to sexual violence, rape, condom failure, and instances of police officers’ misconduct, were mentioned. These led to concerns of anxiety about contracting HIV through rape and unprotected sex [5, 8–10]. Furthermore, FSWs expressed their conviction that clients deliberately perforate or break condoms with the goal of transmitting sexually transmitted infections (STIs) to them [5, 8]. As observed in similar research, sex workers frequently encounter instances of physical, sexual, and verbal aggression. Numerous studies conducted within the environments of many sex workers identified violence as a common occurrence [1, 9, 11, 12, 34, 39, 40, 50]. Consequently, physical assault, coercion, and refusal to accept protection, especially from high-profile officers, were noted as significant health risks to sex work [42, 43, 68]. Recent research indicates elevated levels of violence in some countries. For instance, in the Gambia, police officers reportedly harassed FSWs in the line of work and during patrol operations, resulting to health problems including trauma [45]. Acts of violence and compromised sexual activities were also reported in the advanced countries. In the United Kingdom, 75% of FSWs suffer violence, which accounts for around two-thirds of the total population [56]. Physical abuse and compromised sexual protection primarily contribute to the prevalence of health issues among sex workers [25, 40].

Female sex workers faced multiple human rights violations. The results have shown that issues of discrimination and class concerns existed and how women’s bodies were turned into commodities for distinct ulterior motives. Indeed, increased criminalization and social stigmatisation, potentially leading to further discrimination from health providers, hindered their access and utilisation of health facility care [4, 9, 34–36, 58]. It is critical to understand that barriers to healthcare, such as a lacklustre healthcare system and negative social perceptions about sex work, have a significant impact on the health-seeking behaviours of FSWs, including adherence to treatment plans and continuity of care when they eventually sought care [4, 9, 34–36, 58]. Legislation and enforcement must be revisited to safeguard the rights of female sex workers to prevent illegal abuses to their rights and freedoms. Adequate support systems to enable FSWs to carry out their work without fear or intimidation from state security agencies and healthcare providers is necessary to restore sanity and integrity to the industry. Indeed, continued maltreatment and violations to FSWs endangers the lives of not just their clients but the general population.

Finally, the study found that FSWs felt compelled to use healthcare, aligning with Andersen’s healthcare usage model. In nearly all of the included articles, FSWs showed increased health awareness and self-reported health screening in locations such as Mainland China [5, 8, 46]. Others sought early medical attention when they suspected illness [8]. In fact, the findings revealed that FSWs who had attained secondary and higher levels of education were more conscious about seeking healthcare and in ensuring safe sex. As a result, stakeholders in the health sector, across low- and middle-income countries must promote regular health check-ups, increased population education (especially, because of the low numbers of formal education among women in some countries), and safe-sex practices and behaviours in order to prevent unnecessary morbidities that could endanger the community and sex workers. This is because the findings indicate that a wide range of customers and high-profile persons engage the services of sex workers. It is very appropriate and timely to create ways and laws to protect and ensure FSW access to suitable health care. The findings have shown that stakeholders should advocate for the elimination of exploitation of FSWs seeking care in order to encourage positive healthcare-seeking behaviours among them [1–7, 9, 10, 13, 15–17, 20, 30, 32, 34, 39, 40, 46, 48, 49, 62, 64, 68]. Overall, the report acknowledges that the environment of sex work has shifted over the last two to three decades, notably in several upper-middle and high-income countries [48, 68, 75]. As a result, sex work experiences and health-seeking behaviours would differ from those found in low- and middle-income countries.

### Strengths and limitations of the study

Key strength of the study stems from its geographical coverage and the wide duration which allowed saturation of data on the themes. The study also included articles from The study was guided by Anderson’s healthcare utilisation model; meanwhile different frameworks were used to analyse female sex workers experiences and health seeking behaviours in the included studies. The subject investigated has cultural and health implications, and thus, the non-inclusion of cultural nuances in the search may have left out relevant articles. However, given the saturation of the findings in the themes, it is believed, additional articles may only support what has been found.

Despite the novel findings presented in this study, they must be read against the backdrop of the following limitations. The review investigates the experiences of female sex workers, and their health-seeking behaviours in low-and middle-income countries (LMICs). However, it does not (and cannot) address all of the concerns with geographical distinctiveness in low- and middle-income countries in one study. Further, some articles were not available in full text or freely accessible via search engines for inclusion in the data set. However, the anticipated effect of non-access to the full texts, which are less likely to be published in low impact factor journals, may be to add only a number of supporting articles to the already established themes.

Finally, female sex work has shifted from traditional brothel-based sex work to internet-based sex work and a mix of different types, over the last years. The vulnerabilities faced by women in sex work depend on the type of sex work they are engaged in, whether it’s brothel-based, street-based, massage parlour-based, internet-based, or other forms of transactional sex. However, this study could not explore these nuances, which has limited the conclusions relative to the vulnerabilities across the broad spectrum of female sex work. This study recommends further studies in these areas for policy, social intervention and health programming.

### Implications of the review in relation to anderson’s model

The review’s findings validate several notions outlined in Anderson’s health seeking model, specifically that socio-demographic factors like education, occupation (particularly sex work), and the effectiveness of medications or pharmaceuticals are inadequate for determining the utilisation of conventional healthcare facilities. Nevertheless, the utilisation of orthodox health facilities by FSWs was not significantly influenced by age or marital status. The study also discovered a noteworthy relationship between the vocation of sex work and the practice of self-medication. Variables such as age, education, marital status, work status, and work experience did not show a significant correlation with self-medication from the literature. The findings have shown that female sex work in low- and middle-income countries (LMICs) presents complex policy challenges with significant implications for public health, human rights, and social welfare. Overall, policies regarding female sex work in LMICs should prioritize the rights, health, and well-being of sex workers while addressing the broader social, economic, and structural factors that shape their experiences.

## Conclusion

Complex challenges rooted in economic vulnerability, social marginalisation, and limited access to healthcare services was found to negatively impact female sex workers in low-and middle-income countries (LMICs). It was found that female sex work was motivated by the need to earn living although others engaged in the business for the love it. Nevertheless, FSWs were exposed to multiple risks of sexually transmitted infections, threats of sexual and physical violence against them, as well as human rights violations from clients and law enforcement agencies. Indeed, access to health facility for healthcare become problematic due to discrimination and stigma by healthcare providers. The maltreatment and stigma meted to FSWs, potentially inhibits their health outcomes, the countries attainment of universal health coverage goals, and without access to care for FSWs, HIV could become an endemic health problem. Comprehensive, rights-based strategies that address structural injustices and empower these women to live healthier, more secure lives are necessary to address their special needs. Furthermore, to address the factors relative to healthcare seeking behaviours of FSWs, there is an urgent wake-up call to ensure comprehensive, non-discriminatory, and accessible healthcare is available to improve their healthcare utilisation.

## Data Availability

The Manuscript is a review hence all articles extracted are online and also found under the list of references.
